# Scoping Review of Kaizen and Green Practices: State of the Art and Future Directions

**DOI:** 10.3390/ijerph17218258

**Published:** 2020-11-09

**Authors:** Lidia Sanchez-Ruiz, Beatriz Blanco, Juan A. Marin-Garcia, Elsa Diez-Busto

**Affiliations:** 1 Business and Management Department, University of Cantabria, Avda. Los Castros s/n, 39005 Santander, Spain; blancob@unican.es (B.B.); elsa.diezbusto@unican.es (E.D.-B.); 2 Research Group of Health Economics and Health Service Management, IDIVAL (Instituto de Investigación Sanitaria Valdecilla), s/n, Calle Cardenal Herrera Oria, 39011 Santander, Spain; 3 ROGLE, Department of Organización de Empresas (Business and Management), Universitat Politécnica de Valéncia, Camino de Vera s/n, 46022 Valencia, Spain; jamarin@omp.upv.es

**Keywords:** kaizen, continuous improvement, green production, clean production, sustainability, systematic review, PRISMA

## Abstract

Given the importance that environmental management is acquiring, the main aim of this work is to know what the state of the field kaizen and green practices is at present. A systematic narrative review is conducted in accordance with the PRISMA Statement. Two databases (Web of Science and Scopus) were searched. Finally, after applying the defined inclusion and exclusion criteria, 19 documents were analyzed. Based on the results, it might be concluded that, despite the growing interest in the relationship between kaizen and green practices, this is a topic in the early stages of development, with a clear predominance of case studies. It is, therefore, necessary to develop more research on this kaizen and green issue as improving environmental management is undeniably becoming a must in today’s competitive environment. For instance, more research is needed on the application of kaizen tools as results obtained so far seem not to be conclusive. Additionally, more academic and rigorous studies should be developed on this topic as many of the analyzed papers seem to be clearly created for dissemination among practitioners, some of them lacking the traditional academic structure and scientific method during their development.

## 1. Introduction

Sustainable development has been defined as “*development that meets the need of the present without compromising the ability of future generations to meet their own needs*” [1].

Researchers and practitioners agree with the idea that, in order to achieve sustainable development, first, sustainable organizations must exist [2]. Traditionally, companies have had to offer a quality product/service at the lowest possible cost in order to be competitive and stay in the market. At present, that does not seem to be enough, though. Now, society requires companies to be sustainable, reducing their negative environmental and social impact.

Therefore, due to the increasing competition together with the stricter environmental regulation and legislation, firms have had to adopt new strategies that allow them to deliver quality products/services while increasing effectiveness, reducing waste and costs and being environmentally responsible [3,4].

As a result, the sustainability concept emerged. Initially, sustainability was clearly linked to environmental aspects, but it has now acquired a more holistic vision and is considered to have three main pillars: economic, environmental and social sustainability [5,6,7,8,9]. This is what Elkington [10] first coined as the “triple bottom line” and these three dimensions have been commonly accepted. Recently, Lozano et al. [11] proposed a fourth dimension, time.

Throughout the literature, the environmental dimension, also known as the green dimension [8], has been widely studied [12], probably due to the escalating deterioration of the environment [13]. Due to the lack of a commonly accepted definition of green management [14], several terms have been used in relation to this concept throughout the literature: sustainable management, green management and environmental management [12,15].

In general, green management focuses on the environment and perceives waste as the extraction, and the disposal, of resources at rates or in forms beyond that which nature can absorb [16]. In the same line, Siegel et al. [9] define green as a philosophy and operational approach that enhances the ecological efficiency of an operation, reduces the negative environmental impact of a service or product and maintains or improves financial performance [17]. Thus, green ranges from reactive monitoring of the general environment management programs to more proactive practices [13]. 

Although it is true that initially the adoption of green strategies was seen as an imposition, perceptions are changing, and more companies see it as an opportunity [6]. It is observed that more and more stakeholders are asking for more responsible organizations with respect to their products and/or processes [18,19]. 

Regarding the concepts clean and green, the vast majority of authors consider them as synonyms. Only some authors, like Cagno et al. [20], consider clean practices to be included in and more specific than green. 

In this study, as the main stream, we understand green and clean production as synonyms which, in turn, are included in a wider concept: sustainability. 

Considering the existing need to combine economic benefits, operational efficiency and sustainability, it was considered whether it was possible to improve the environmental performance of companies through the application of philosophies or management systems traditionally focused on the improvement of processes (efficiency) and the client (effectiveness) [21]. For example, along this line, the literature shows a growing number of studies aimed at analyzing the relationship between lean management and green management [17,22,23,24].

In parallel, there would be the green kaizen movement, which is the focus of this study. Although there seems to be some confusion about the relationship between both concepts (lean and kaizen), without being exhaustive since this is not the object of our study, it should be mentioned that the concepts, although related [25,26], should not be treated as synonyms [27]. Each of them has its nuances and characteristics, so it seems appropriate to treat them separately.

Overall, the ultimate goal of this review is to know what the state of the field kaizen and green practices is at present. Thus, it is considered that a scoping systematic review is the most suitable approach to achieve it. Scoping reviews provide an initial indication of the potential size and nature of the available literature on a particular issue [28]. Similarly, systematic reviews are aimed at identifying, appraising and integrating the evidence about a specific topic in order to inform practice, policy and future research [29,30].

Specifically, the research questions stated in our study are the following:

- Which objectives and research questions do the papers respond to?
- Do theoretical or empirical papers predominate? What are the main methodologies applied? What are the most common sample sizes?
- Concerning the empirical studies, which economical activities and countries have they been applied to?
- What are the main conclusions achieved?
- What are the future lines of research stated by the studies developed so far?

## 2. Materials and Methods 

This systematic review provides an indication of the size of the available literature on kaizen and green production. It also examines the extent of research and identifies research gaps. It was conducted and reported in accordance with the PRISMA (Preferred Reporting Items for Systematic Reviews and Meta-Analyses) Statement [29,31].

### 2.1. Inclusion and Exclusion Criteria

The first step is the identification of the existing research papers which relate kaizen and green or clean production. Throughout this section, we describe the different phases that have been followed in the search [32,33].

First of all, the inclusion criteria used were:

- Document type: research paper or conference paper;
- Publication language: English or Spanish;
- Period: all years available until 31 December 2019;
- Topic: kaizen and clean/green production.

On the other side, documents were excluded if they did not meet the inclusion criteria and/or they met the exclusion criteria:

- Understanding sustainability as maintaining kaizen practices through time (opposite to the “honeymoon effect”) instead of environmental sustainability (green or clean production). For instance, this is the case of the work of Todorut et al. [34].

### 2.2. Search Strategy and Study Selection

Two electronic databases (Clarivate-Wos and Scopus) were searched. Research papers and conference papers (proceedings) written in English or Spanish were included in this review. Text words and indexed terms like “kaizen, clean, green” were included in the search terms. Table 1 shows the complete search strategy used for this review.

The 57 identified articles were imported into Mendeley software (manufacturer name, city, country). Twelve duplicates were removed and the title and abstract of the 45 remaining papers were screened. Two authors (LSR and BB) independently screened the titles and abstracts of those 45 papers and excluded those which did not meet the inclusion criteria. After that, the independent results of both authors were compared based on the decision matrix shown in Table 2.

After the initial independent evaluation, authors 1 and 2 met to solve the differences. Twenty papers were included and eighteen were excluded by mutual agreement, but disagreement persisted on 4 papers (author 1 considered they should be included, and author 2 considered they should be excluded). Those four papers together with the three papers classified as “double doubt” were selected for an additional analysis by author 3 (JAMG). Finally, only one of the 7 undecided papers was included (Appendix A).

In order to calculate the inter-rater indexes, the three “double doubt” papers were considered as excluded to maintain two categories. Several inter-rater agreement indexes were calculated (observed agreement = 0.85; Cohen’s kappa = 0.53; bias-adjusted kappa = 0.70 and Krippendorff’s alpha = 0.43). Analyses were performed using the epi.R package v. 0.9–99 and irr v.0.84.1 for R version 3.6.1 (Mark Stevenson, Parkville, Australia). Considering the cut-off values [35], the agreement between author 1 and author 2 can be considered substantial.

As the final number of papers was 21, it was decided to widen the search with more terms. Therefore, author 3 (JAMG) independently conducted a new search. The inclusion criteria were the same, but the number of search terms was increased, as it can be seen in Table 3.

As expected, the number of results was higher than in the previous search as it also included “sustainab*” as a new term. However, once author 3 screened titles and abstracts checking that the papers met the inclusion criteria, the final number was dramatically reduced: 15 documents for Scopus and 4 documents for Web of Science. Among the 19 documents, 3 were duplicates, so the final sample in this case is 16 documents. Fourteen of those 16 papers were already included in the first search strategy conducted by authors 1 and 2, and only 2 were new [36,37].

Overall, 23 papers were selected to be included in the review (21 from the first search performed by authors 1 and 2, and 2 new papers identified through the second search performed by author 3). The full text was available for 20 documents. One of the documents [36] was excluded after reading the full text as it met the exclusion criteria.

**Figure 1:**
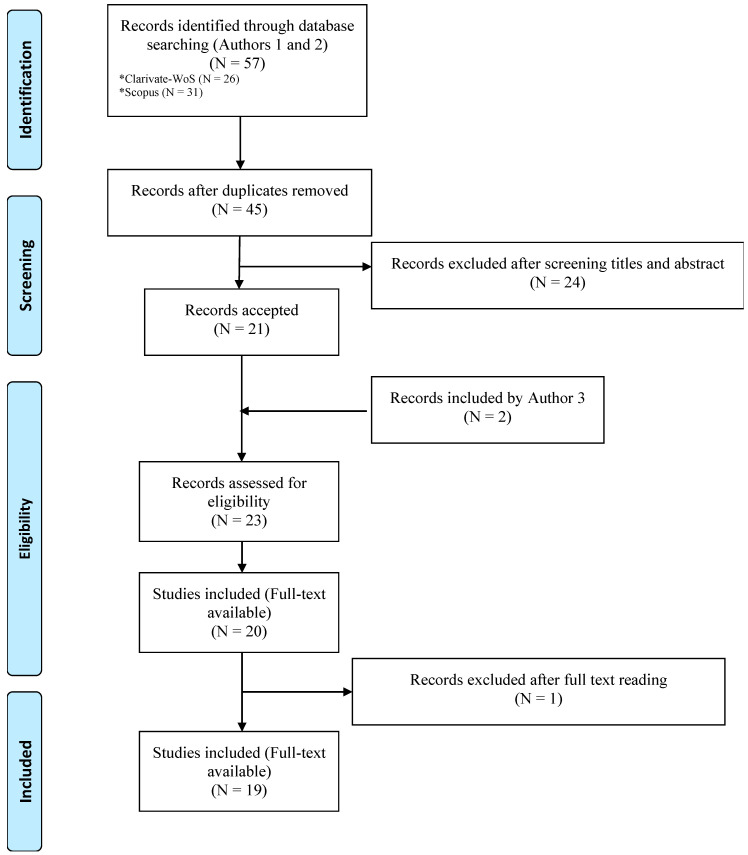
Flow chart of the study selection protocol.

The whole process is described in Figure 1.

### 2.3. Data Synthesis and Analysis

Once the final sample of documents has been identified, content analysis must be conducted. AtlasTi software (ATLAS.ti Scientific Software Development GmbH, Berlin, Germany) was used in this case. A set of codes was developed and agreed by the authors in order to collect the information from all the studies. The selected codes respond to the research questions set in the Introduction section. Table 4 includes the established codes that will be used. In addition, some coding examples for each of the codes are shown in Appendix B. It should be noted that the code “False Positive” will be assigned to those studies that, after a thorough review, do not meet the inclusion criteria or meet the exclusion criteria discussed above. 

For the coding process, the first two authors (LSR and BB) will independently code the 19 articles. Once the codification is finished, the four authors will independently analyze the coded text fragments. After the independent analysis, a common work session will be held to draw the final conclusions of the study.

## 3. Results

Throughout this section, the main results obtained from the coding and content analysis are described. Specifically, each of the subsections will answer one of the research questions posed.

**Figure 2:**
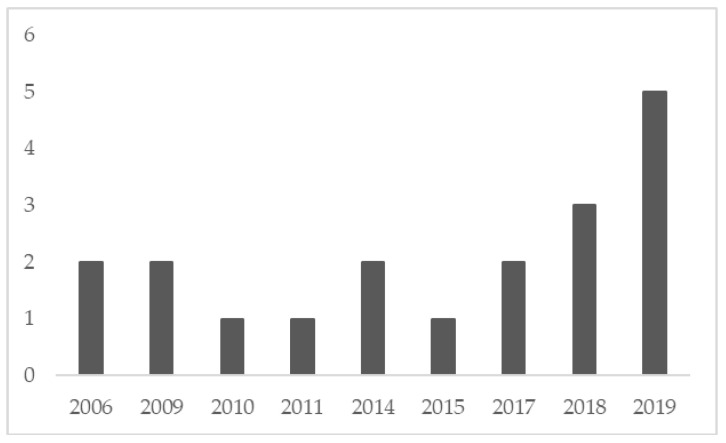
Number of publications per year.

Before starting, as an overview, Figure 2 shows the number of publications per year. The number of published papers related to the subject has been rising since 2015. Before this year, there is a period in which the number of publications follows a flat trend with continuous ups and downs.

### 3.1. Which Objectives and Research Questions Do the Papers Respond to?

Among the nineteen articles analyzed, fifteen are case studies that show concrete applications of kaizen with a clear orientation towards environmental sustainability [4,37,38,39,40,41,42,43,44,45,46,47,48,49,50]. In all of them, the aim is to show the synergies between both kaizen and green philosophies. To measure these synergies, the works measure the impact of kaizen on certain environmental indicators, for example: energy consumption, resource use, CO2 emissions, carbon footprint and waste generation. 

Although there is this common purpose in all of them, the approaches are subtly different. Some articles consider that the mere application of traditional kaizen techniques can already lead to an improvement in environmental indicators. In others, however, the authors highlight the need to rethink the operations management philosophy and orient it to the green philosophy, which in many cases is considered the next stage.

Another differentiation between the analyzed cases is that in some, continuous improvement is treated as an integral philosophy [8], while in other cases, the application and adaptation of certain tools is discussed [50].

As a noteworthy aspect, it should be noted that the work of Bellgram et al. [47] is the only one that refers to the importance of human resources in this type of change initiative. Specifically, the work tries to analyze how to “hook” the operators and managers to get involved in the process.

The remaining four articles, that are not case studies, have a more theoretical aspect. Thus, the work by Zokaei et al. [51] is a literature review, although not systematic, on how operational goals are aligned with green. On the other hand, the work of Cherrafi et al. [52] proposes a model/framework for running improvement workshops composed of five stages: prepare for the event, conduct the event, combine and organize ideas, decide on improvements and track and preserve the results. Finally, the studies carried out by Pampanelli et al. [8,53], in addition to including a model, present an application of their model in an engineering corporation and in an automotive production plant. Through their model, the authors intend to adapt the principles of continuous improvement, providing them with a greater environmental orientation.

### 3.2. Do Theoretical or Empirical Papers Predominate? What Are the Main Methodologies Applied? What Are the Most Common Sample Sizes?

As has already become apparent in the previous section, research on the subject is very scarce. Despite this fact, there seems to be a clear predominance of empirical studies and, more specifically, of case studies. Only three studies incorporate the proposal for a model and only one includes a review, although it is not systematic (Table 5).

With regard to data collection methodologies, many of the works do not specify the technique used. In some cases, however, express reference is made to individual or group interviews [39], surveys [4,42] and brainstorming sessions [47].

On the other hand, the analysis methodologies used include techniques such as descriptive analysis [4], modeling and simulation [48], structural equations [4], qualitative analysis methods such as content analysis [41], correlations [50] and ANOVA [42].

Finally, with regard to the sample sizes in the studies, a clear predominance of cases based on a single company is perceived. It is true, however, that some studies focus on a certain sector or the entire supply chain, therefore including information from several companies. Only the studies that incorporate the survey as a collection sample have larger sample sizes: 239 [4] and 250 [50].

### 3.3. Concerning the Empirical Studies, Which Economical Activities and Countries Have They Been Applied to?

There is wide variability in the sectors in which the studies are carried out (Table 6), although there is a clear predominance of the manufacturing sectors. On the contrary, the work of Hussain et al. [4] is applied to the service sector, specifically to a group of hotels.

Likewise, it is important to note the work of Cherrafi et al. [52], since it is the only one that includes companies from different sectors in its sample (aerospace and automotive). This is because the authors consider validating their model in different settings.

On the other hand, with regard to the geographical distribution of the studies, it should be noted that this information is not specified in all of them. When reference is made to it, a wide variability is again perceived, and no pattern or area can be found that is at the fore in this subject (Table 7). Although it is true that several studies were carried out in the US [42,49,52], there were also projects in Europe (UK [44], Romania [38], Italy [45]), Asia (Thailand [41], United Arab Emirates [4]) and South America (Brazil [8]).

### 3.4. What Are the Main Conclusions Achieved?

One of the first conclusions derived from the studies is the growing interest in the relationship between kaizen and green, especially since 2009. In the opinion of Zokaei et al. [51], this movement is right now where the movement of quality was 40 years ago. At that time, quality was understood as an extra cost, an opposite objective to profitability. With the passage of time, it was seen that more quality implied more value and that doing things right the first time meant a reduction in costs. Now, the same is happening with sustainability and environmental aspects. There are many companies that consider environmental objectives to be at odds with cost objectives, the same perception that existed with quality in past decades [51]. As with quality, with the passage of time, it has been proven that there is no dilemma between sustainability and cost. It is high time that sustainability is seen as an opportunity and not as a cost. On the other hand, it must be considered that, as Zokaei et al. [51] point out, environmental management can now be a competitive advantage, but the future will be a “must have”.

Zokaei et al. [51] also proposed the term total environment management, in a clear reference to the commonalities between quality and environmental management. They suggest that the latter can borrow many ideas in terms of structures and methods of quality management that, undoubtedly, has a much longer history.

Pampanelli et al. [8,53] proposed a model which adopts a kaizen approach. Specifically, Pampanelli et al. [53] suggest moving from the traditional quality, delivery, cost (QDC) to QDCE (also incorporating “environment”). For instance, in operational terms, a new type of waste could be incorporated in traditional waste: environmental. In this way, the three core sustainability dimensions: profit, people and planet, would be worked on.

More specifically, some of the analyzed studies show how there are certain tools traditionally associated with kaizen that can be successfully applied to environmental issues. For example, in the work of Ramakrishnan et al. [48], a value stream map (VSM) is applied. Instead of using inventory level or time consumption to identify areas for improvement, energy consumption levels are incorporated. 

It is true, however, that there are no conclusive results concerning tools’ application and their usefulness for environment management, as studies differ. Garza-Reyes et al. [50] conclude that the impact of the tools is uneven. According to the authors, the application of kaizen/continuous improvement only showed an effect on the use of materials and release of pollutants. In the same vein, the work of Alves Pinto Junior and Mendes [40] concludes that the use of some tools such as the Poka-Yoke and the Ishikawa diagram, or the application of the Plan, Do, Check, Act (PDCA) cycle are useful when reducing consumption of energy and water.

Another of the main conclusions drawn from the analyzed articles is that the application of kaizen and green generates direct benefits of various kinds for companies, for example: cost reduction derived from lower energy consumption [48], higher productivity in the use of natural resources [8] or a reduction in emissions without reducing the quality of the product/service offered [37]. Likewise, indirect environmental benefits are perceived, for example, by reducing transport, fewer emissions are achieved [47]. In this line, the conclusion drawn by Hussain et al. [4] is interesting. These authors, through a correlation analysis, conclude that, just as continuous improvement practices have a greater impact on economic indicators, green practices have it on environmental indicators. Therefore, it seems logical to think that to achieve a balance, it is necessary to unite both philosophies and obtain synergies.

Finally, in a different vein, two additional ideas stand out that are worth mentioning. The first one is the importance of human resources in this type of change initiative. Bellgran et al. [47] emphasize the need for management support, as well as the commitment and positive attitude of employees. The second idea, included in the works of Goyal et al. [37] and Pampanelli et al. [53], is that large investments are not necessary to reduce the environmental impact of a company. Both studies consider it important to put an end to this myth since large investments represent a barrier to companies that dare to work on reducing their environmental impact. While it is true that these improvements could come from the hand of reengineering, that undoubtedly requires large investments, it is important to show that there are other possible alternatives, more economical, kaizen being one of them.

### 3.5. What Are the Future Lines of Research Stated by the Studies Developed So Far?

There are several lines of research that are suggested in the analyzed studies. In the first place, and despite the number of case studies identified, the authors point out the need for more practical studies that demonstrate the existing synergies between kaizen and green. In addition, it is also proposed to study whether companies that apply kaizen and green simultaneously obtain better environmental results than those that apply only one of the techniques [51].

It is also suggested to work on more theoretical models that, synergistically, combine the ideas of both philosophies (non-value-added activities removal and green), developing the idea of total environment management more precisely [51].

Finally, as lines of work to be developed, it is pointed out the need to carry out more studies in service sectors such as health or banking [4,48], as well as increasing the scope of the studies. These are mostly focused on a single company (even a department or area of the same), when in reality it would be interesting to broaden the perspective and cover the entire value chain [8]. 

## 4. Discussion

Based on the obtained results, it might be concluded that, despite the existing homogeneity among the analyzed articles, with a clear predominance of case studies, it is difficult to draw generalizable conclusions since these are very specific application cases with a clear vocation for dissemination among practitioners, with some of them lacking the traditional academic structure and scientific method during their development. Without a doubt, their usefulness is undoubted since they serve as a model for other companies and because they allow us to see common aspects to take into account. That said, it would be necessary to go one step further to achieve the development of a firm theory in the field of kaizen and green/clean practices. Although it is true that three of the articles found make an attempt to propose a model, they follow the same framework as previous models do (PDCA), the Key Performance Indicators (KPIs) being the main and unique difference. Therefore, more research should be developed in this respect, and one of the questions that might be answered in the future is how companies might manage to be green. A future research line will be proposed in the final paragraphs of this section in this respect.

This may be due to the fact that the intersection of both philosophies is still a topic in the early stages of development [51]. There are several results that support this statement. Firstly, the high percentage of case studies and the almost total absence of more elaborate theoretical works is also indicative that we are facing an expanding field. Finally, the fact that most studies focus on industrial/manufacturing economical activities suggests that this particular topic might be in its initial phase. It could be that, as happened with other management systems (mass production at the beginning of the 20th century or the lean philosophy at the end) which were first applied in manufacturing (especially in the automotive industry), kaizen–green practices may have been first applied in manufacturing environments and, over time, the number of studies based on service industries will increase. In this sense, it would be interesting to know which economical activities are the ones that produce the highest CO2 emissions so that efforts are focused on them. Thus, the highest impact will be achieved.

From all the above, it might be concluded, as other authors pointed out, that it is necessary to develop more research on this kaizen and sustainability issue that allows obtaining generalizable conclusions [4,8,48,51].

Another interesting idea regarding the analyzed case studies is that they are all success stories. It is hard to believe that there has been no case of implementation with unfavorable results and that the synergies between kaizen and green/clean initiatives have always been clear. In the authors’ opinion, the publication of failure cases should be encouraged as they serve as an example of what not to do. Of course, these must be well informed and grounded.

Regarding the relationship established between kaizen and green/clean management, there are several aspects to highlight. In the first place, it is striking how, although our search strategy used the kaizen concept, there are many studies obtained that are about lean management. This leads us to wonder if both concepts are understood as synonyms, although, in theory, they are not. The need to clarify these concepts and the relationship between both has already been evident in previous studies [27]. Therefore, and although it is not the objective of this work, it seems that a greater theoretical reflection is necessary to end the confusion of existing terms.

Secondly, another reflection is that in all the analyzed articles, the relationship between kaizen and green/clean management is given by the fact that the application of improvements results in a reduction in waste associated with environmental impact. However, in the opinion of the authors, this orientation should be enriched, and the relationship should also be established from the point of view of value. In other words, environmental improvements should not be understood as a consequence of kaizen, but as an objective. More and more customers are already demanding, as a sign of value, that companies’ processes are sustainable. A clear example of this is the B-Corp certification that is only given to those companies that, after an audit process, prove to be economically, socially and environmentally sustainable [54,55,56,57,58]. These companies are a clear example of how environmental objectives can be incorporated into the mission of the company from the beginning, not only as a result of the application of improvements in the process.

What does seem clear is that the environment has become another key business variable, in addition to quality, service and cost. The question is, are they compatible or conflicting objectives? Is it a question of priorities? If the priority is cost, the company will ask what its best environmental performance is given this cost limit. However, if the environment is the priority, the question will concern what the cost will be, given certain environmental goals. The big question is, can a situation where these objectives are not in conflict be reached? In this sense, and as Zokaei et al. [51] highlight, it would be advisable to learn from the trajectory of the total quality management movement which concluded that quality and cost were not conflicting objectives.

From a more operational point of view, the results on the application of kaizen tools do not seem conclusive. Therefore, it would be necessary to carry out more studies in this regard. If kaizen and green/clean practices are to be applied synergistically, can the tools be applied in the same way? Are adaptations needed? Of course, the analysis of the tools and their application must be preceded by the necessary theoretical reflection that has already been commented on in previous paragraphs.

Based on the above, as a future research line, it would be interesting to compare whether there are differences in how continuous improvement is applied between cost-driven and environmental-driven companies. If, when comparing their vision, it is concluded that their perspective is the same, the application of the same tools could be recommended. If, on the other hand, the perception is not the same, it would not only be necessary to change the focus (from cost to sustainability), but also the way of applying the tools. As a concrete example, a study between B-Corp companies could be carried out. Specifically, it would be interesting to know the case of a company that, having practiced continuous improvement habitually, has been certified as B-Corp. It could be analyzed whether it still has the same vision of continuous improvement, or if it has changed, one could check what the changes have been.

On the other hand, continuous improvement is a cornerstone of other operations management paradigms (such as Total Quality Management (TQM), Total Productive Maintenance (TPM), Lean, Agile …). Therefore, it would be interesting to investigate the relationship of these paradigms with clean production and compare those results with the specific results related to kaizen that have been presented in this work. Thus, it could be distinguished whether this relationship is due specifically to kaizen, as analyzed here, or to the whole set of practices and pillars that integrate other broader paradigms.

Finally, as mentioned above, it would be necessary to develop more research on how a company can become green. In our opinion, three ways are possible: randomly (not recommended), implementing changes through reengineering or implementing changes through kaizen.

## 5. Conclusions

The objective of this study is to know what the state of the field kaizen and clean-green practices is at present. To achieve this, a systematic literature review has been carried out.

Regarding the first research question, which objectives and research questions the analyzed papers respond to, the results obtained seem to show that kaizen can be an interesting philosophy to achieve environmental sustainability, which must be incorporated as an additional objective. Thus, the analyzed articles show how synergies can be obtained from the joint application of kaizen and green management techniques. 

At this point, it is important to note that, as indicated in several of the articles reviewed, environmental objectives are becoming increasingly important, ceasing to be an option and becoming a must-have. In fact, in the future, other objectives (cost, for instance) may have to be subordinated to environmental KPIs. As a result, the research perspective might also be changed. So far, environmental improvements have been analyzed as a consequence of applying kaizen; henceforward, environmental objectives should be included as an input for kaizen. They are not a consequence but a requisite.

Concerning the type of studies and the methodologies applied, based on the results, it might be seen that empirical studies predominate, especially case studies. Many of them do not inform about the method they used when collecting the data for their analysis (action research, survey, interview, KPIs analysis ….). Therefore, we recommend authors to include this information in their future research works. On the other side, more qualitative studies should be carried out, spreading the research on this field to other sectors and geographical areas.

In this line, as seen in Figure 2, it can also be concluded that it is a field of growing interest for both academia and industry, as shown by the clear informative orientation of many of the analyzed articles. It is true, however, that it seems necessary to extend empirical research to other sectors, and to carry out more theoretical research to obtain generalizable conclusions.

From an operational point of view, it is essential to investigate whether the tools traditionally associated with kaizen are useful for fostering green/clean initiatives and how they should be applied. The results obtained so far, although interesting, are limited and, in some cases, contrary.

All in all, based on the main conclusions and future research lines stated by the analyzed papers, it might be said that the kaizen–green topic is a developing and very promising field in which further research is required. As mentioned above, it is necessary to develop more research on this topic in order to obtain generalizable conclusions.

Finally, regarding the future research lines, concerning the empirical studies, two points should be highlighted. On the one side, we have realized that most of the case studies do not inform about the method they used when collecting data (action research, survey …). Therefore, we recommend future authors to include this information in their research works. On the other side, it is recommended to develop more quantitative studies, spreading research to other sectors and geographical areas.

Overall, the main contribution of this paper is that it develops a rigorous literature review about kaizen and green/clean terms. In fact, as far as the authors are concerned, this is the first review that relates both concepts. Similarly, we consider that this paper is useful for academics interested in this field of research. First, it shows that the topic kaizen–green is in its early stages and more research should be develop. Secondly, the main research works of the field are identified and commented on. Finally, throughout the Discussion and Conclusion sections, several recommendations and future research lines have been proposed. 

On the other side, we believe the content of this study might be of interest to practitioners. Our results might show them that the green movement, as it happened with quality in the previous decades, is becoming a key issue. In fact, environmental objectives will probably become a must in the near future. Thus, we hope that this paper has raised practitioners’ awareness of the relevance of this topic.

This work has two main limitations. In the first place, the search was based on two databases: Web of Science and Scopus. Although it is true that both bring together the largest number of articles on the subject, their scope in conference proceedings is more limited. Therefore, in future reviews, it would be interesting to incorporate other databases such as EBSCHO or ABI. Secondly, the number of publications found is limited, a sign of the incipient development of the topic. In this sense, it would be advisable to repeat this analysis in a period of three to five years. By that time, new works that are currently under development will have been published.

## Figures and Tables

**Table 1:** Search strategies by authors 1 and 2.

Database	Search Strategy	Results
Clarivate-Wos	TOPIC: (kaizen) AND TOPIC: (green OR clean) Refined by: TYPES: (ARTICLE OR MEETING) AND LANGUAGES: (ENGLISH OR SPANISH) Timespan: 1900–2019. Databases: WOS, CCC, DIIDW, KJD, MEDLINE, RSCI, SCIELO.	26
Scopus	TITLE-ABS-KEY (kaizen AND (clean OR green)) AND PUBYEAR <2020 AND (LIMIT-TO (DOCTYPE, “ar”) OR LIMIT-TO (DOCTYPE, “cp”)) AND (LIMIT-TO (LANGUAGE, “English”) OR (LIMIT-TO (LANGUAGE, “Spanish”)	31

**Table 2:** Decision matrix.

		Author 1 Decision about Paper		
		Include	Doubt	Exclude
**Author 2 decision about paper**	**Include**	Included	Included	Undecided
	**Doubt**	Included	Undecided	Excluded
	**Exclude**	Undecided	Excluded	Excluded

**Table 3:** Search strategies by author 3.

Database	Search Strategy	Results
Clarivate-Wos	TOPIC: ((kaizen) and (clean or green or sustainab*)) Timespan: 1900–2020. Indexes: SCI-EXPANDED, SSCI, ESCI.	88
Scopus	TITLE-ABS-KEY (kaizen AND (clean OR green OR sustainab*))	156

**Table 4:** Codes.

Code	Definition
Objective	The objective/aim/research question stated by the authors of the study.
Review	Literature review study (systematic or not) or bibliometric analysis
Theoretical	Theoretical study. It reviews or critically analyzes a concept and/or its evolution over time, but it cannot be classified as a literature review.
Empirical	Study that includes some type of empirical analysis (case study, survey, interview …).
Geo	Region(s) or country(ies) where the empirical study is conducted.
Sector	Sector(s) where the empirical study is conducted.
Size	Size of the final sample on which the empirical study is based.
Method for collecting the data	Method used for collecting the data (survey, interview, secondary sources…).
Method for analyzing the data	Method applied to analyze the data (regression, factorial analysis, ANOVA, structural equation modeling …).
Conclusion	Main conclusion(s) of the study.
Future lines	Future research lines suggested by the authors of the study.

**Table 5:** Typology of the studies.

Typology		Number of Papers
Empirical studies	Case study	15
Theoretical studies	Literature review	1
	Model proposal	3

**Table 6:** Sector distribution.

Authors	Sector
Acharry et al. [41]	Electrical/Electronic
Alves Pinto Junior and Mendes [40]	Electronic
Antomarioni et al. [45]	Manufacturing
Bellgran et al. [47]	Pharmaceutical
Birkie et al. [39]	Pharmaceutical
Cherrafi et al. [52]	Aerospace and automotive
Garza-Reyes et al. [50]	Manufacturing
Goyal et al. [37]	Manufacturing
Harris et al. [42]	Automotive
Hughes [49]	Manufacturing
Hussain et al. [4]	Hotels
Nahmens [43]	Housing
Pampanelli et al. [8]	Manufacturing
Pampanalli et al. [53]	Engineering
Ramakrishnan et al. [48]	Manufacturing
Whyle and Bland [44]	Manufacturing

**Table 7:** Geographical scope.

Authors	Geographical Scope
Acharry et al. [41]	Asia (Thailand)
Alves Pinto Junior and Mendes [40]	South America
Antomarioni et al. [45]	Europe (Italy)
Birkie et al. [39]	Europe
Cherrafi et al. [52]	US
Garza-Reyes et al. [50]	World
Goyal [24]	Asia (India)
Harris et al. [42]	US
Hughes [49]	US
Hussain et al. [4]	Asia (United Arab Emirates)
Pampanelli et al. [8]	South America (Brazil)
Pampanelli et al. [53]	South America (Brazil)
Vais et al. [38]	Europe (Romania)
Whyle and Bland [44]	Europe (UK)

## Appendix A

**Table A1:** Article selection matrix.

Article	Author 1 Decision	Author 2 Decision	Author 3 Decision	Final Decision
Acharry et al. [41]	I ^1^	I	-	Included
Aflaki et al. [59]	D	D	E	Excluded
Alves Pinto Junior and Mendes [40]	I	D	-	Included
Antomarioni et al. [45]	I	D	-	Included
Arya and Choudhary [60]	I	E	E	Excluded
Bashkite et al. [61]	D	D	E	Excluded
Bellgran et al. [47]	I	I	-	Included
Birkie et al. [39]	I	I	-	Included
Cherrafi et al. [52]	I	D	-	Included
Garza-Reyes et al. [50]	I	I	-	Included
Guruchannabasavaiah and Latha Shankar [46]	I	E	I	Included
Hammadi and Herrou [62]	I	E	E	Excluded
Harris et al. [42]	I	D	-	Included
Hughes [49]	I	I	-	Included
Hussain et al. [4]	I	I	-	Included
Mishra and Pradhan [63]	I	I	-	Included
Nahmens [43]	I	D	-	Included
Pampanelli et al. [8]	I	I	-	Included
Pampanelli et al. [53]	I	I	-	Included
Maginnis et al. [64]	I	E	E	Excluded
Ramakrishnan et al. [48]	I	I	-	Included
Sondgeroth and Lioi [65]	D	I	-	Included
Vais et al. [38]	I	I	-	Included
Vidmar [66]	D	I	-	Included
Whyle and Bland [44]	I	D	-	Included
Zingale [67]	D	D	E	Excluded
Zokaei et al. [51]	D	I	-	Included

^1^ I: included; D: doubt; E: excluded.

## Appendix B. Some Codification Examples

**CODE: OBJECTIVE**

**2:1 This paper’s purpose is to review how the field of lean and green has…… (2:62 [2:227])-D 2: 23_Zokaei et al. 2017.pdf**

This paper’s purpose is to review how the field of lean and green has been evolving. The authors draw parallels between the fields of sustainability and quality management.

**4:1 The main objective of this paper is to propose a new model, which we c…… (1:1394 [1:1766])-D 4: 7_Pampanelli et al. 2014.pdf**

The main objective of this paper is to propose a new model, which we call the lean and green model. In this model, we integrate environmental sustainability into pure lean thinking. The model presented in this paper adopts a kaizen approach to improve mass and energy flows in manufacturing environments that already possess the necessary deployment level to apply lean thinking.

**5:1 The purpose of this paper is to empirically assess the impact of integ…… (2:372 [2:563])-D 5: 12_Hussain et al. 2019.pdf**

The purpose of this paper is to empirically assess the impact of integrated lean and green practices on the sustainable (environmental, economic and social) performance of a hotel supply chain.

**CODE: GEO**

**6:18 Morocco (9:1885 [9:1891])-D 6: 18_Cherrafi et al. 2019.pdf**

Morocco

**7:11 India (1:2570 [1:2574])-D 7: 28_Goyal et al. 2019.pdf**

India

**14:16 Latin America (9:1271 [9:1283])-D 14: 6_Alves Pinto Junior and Mendes 2017.pdf**

Latin America

**18:3 Telford (UK). (1:420 [1:433])-D 18: 11_Whyle and Bland 2017.pdf**

Telford (UK)

**CODE: SECTOR**

**3:4 high-end server manufacturing facility. (2491:2529)-D 3: 17_Ramkrishnana et al. 2009.docx**

high-end server manufacturing facility

**5:2 A case study of a large sample of UAE hotels is used to collect an…… (2:780 [2:970])-D 5: 12_Hussain et al. 2019.pdf**

A case study of a large sample of UAE hotels is used to collect and analyze empirical data, validate the measurement model and test study hypotheses using structural equation modeling (SEM).

**6:3 The model was validated through two case studies in the aerospace and a…… (1:1635 [1:1725])-D 6: 18_Cherrafi et al. 2019.pdf**

The model was validated through two case studies in the aerospace and automotive industries.

**CODE: SIZE**

**5:16 f 239 respondents were considered in the analy (8:277 [8:322])-D 5: 12_Hussain et al. 2019.pdf**

239 respondents were considered in the analysis

**6:3 The model was validated through two case studies in the aerospace and a…… (1:1635 [1:1725])-D 6: 18_Cherrafi et al. 2019.pdf**

The model was validated through two case studies in the aerospace and automotive industries.

**14:24 single case study (18:1651 [18:1667])-D 14: 6_Alves Pinto Junior and Mendes 2017.pdf**

single case study

**CODE: METHOD FOR COLLECTING THE DATA**

**13:4 The methodology used for this case study entailed observation a…… (5:1872 [5:1955])-D 13: 10_Nahmens.pdf**

The methodology used for this case study entailed observation and interviews

**15:8 team member, 12 in total), observations at the production lines (both…… (4:1 [4:414])-D 15: 5_Birkie et al. 2018.pdf**

Observations at the production lines (both as included in the GPM structured 5-step procedure and as follow-up), individual interviews with selected respondents and group interviews and discussions with the production operator teams and the supportive team (lean manager, lean change agent, production engineers, environmental expert and coordinators, energy experts and production leaders).

**17:11 Two methods were applied for developing these analyses: (1) brainstorm…… (5:316 [5:446])-D 17: 16_Pampanelli et al. 2015.pdf**

Two methods were applied for developing these analyses: (1) brainstorm sessions with participants and specialists and (2) A3 analysis.

**19:6 Surveys (5:1624 [5:1632])-D 19: 9_ Harris et al. 2011.pdf**

Surveys

**CODES: METHOD FOR ANALYZING THE DATA**

**3:10 modeling and simulation techniques (19385:19417)-D 3: 17_Ramkrishnana et al. 2009.docx**

**modeling and simulation techniques**

**5:18 AMOS software package was used for the SEM to study the causative rela…… (8:1001 [8:1169])-D 5: 12_Hussain et al. 2019.pdf**

AMOS software package was used for the SEM to study the causative relations and to test study hypotheses. SEM was mainly performed through confirmatory factor analysis.

**11:2 Quantitative data analysis was carried out with the LISREL co…… (3:4120 [3:4282])-D 11: 8_Acharry et al. 2014.pdf**

Quantitative data analysis was carried out with the LISREL computer program, whereas analysis of qualitative data was carried out using content analysis.

**12:18 The collected data were then subjected to a correlation analysis, which…… (5:1974 [5:2328])-D 12: 21_Garza-Reyes et al. 2018.pdf**

The collected data were then subjected to a correlation analysis, which was performed using the IBM SPSS Statistics software version 23, to investigate the effect of lean methods on environmental performance. To verify the findings of the correlation analysis, a structural equation modeling (SEM) analysis was subsequently performed using the AMOS22 software.

**CODE: CONCLUSION**

**2:3 this paper contributes to the fields of operations management a…… (2:1681 [2:2073])-D 2: 23_Zokaei et al. 2017.pdf**

This paper contributes to the fields of operations management and sustainability by proposing a change in businesses’ mind-set about sustainability. Rather than seeing environmental protection as a cost, it should be regarded as an opportunity for enhancing economic performance. In doing so, we can seek inspiration from the fields of quality management and the total quality movement.

**3:7 This paper presents a framework (Exhibit 2) to identify forms of energ…… (7886:8563)-D 3: 17_Ramkrishnana et al. 2009.docx**

This paper presents a framework (Exhibit 2) to identify forms of energy waste in processes and operations in a supply chain, which can be used in conjunction with the eight types of lean waste. Once the various forms of energy waste are identified, kaizen bursts will be used to represent the green opportunities and then improvement actions will be defined. We represent these bursts as an alphanumeric indicator that signifies the degree of energy consumption and the ease of addressing the opportunity. Once these actions are defined, the framework employs discrete event simulation techniques to quantify the energy savings, along with its impact on addressing other types of lean waste.

**16:10 results achieved allowed demonstrating how, in this applica…… (7:2700 [7:3418])-D 16: 13_Antamaroni et al. 2018.pdf**

Results achieved allowed demonstrating how, in this application, lean practices implementation favors better environmental performances without recurring to any heavy investment. For more accurate monitoring of energy consumption, however, the network should be provided with separate counters for each production cell. In this way, data used for the environmental evaluation could be more precise and the results would provide a global view of the effective sustainability performances. Extending the lean practices event to lithography and drawing and blanking operations will surely contribute to improve operational performances further.

**CODE: FUTURE LINES**

**2:21 Similarly, research needs to be carried out to define “total environmen…… (8:2254 [8:2621])-D 2: 23_Zokaei et al. 2017.pdf**

Similarly, research needs to be carried out to define “total environmental management” beyond conforming to end-of-pipe specifications and in terms of building environmental sustainability into products and processes. This new research is likely to be informed by systems theory, organizational behavior and change management (Argyris, 1999; Senge, 1990).

**4:35 The model presented in this paper was designed for, and is limited to,…… (10:2797 [10:3266])-D 4: 7_Pampanelli et al. 2014.pdf**

The model presented in this paper was designed for, and is limited to, the cell level, which is the first-value stream level of a manufacturing business that supports the principles of lean thinking. This study can be expanded to the other value stream levels, including the second-level flow (i.e., the factory) as well as the third-level flow, or extended value stream, level (i.e., multiple factories or the supply chain). These extensions will be the subject of future studies.

**5:27 Secondly, data have been collected from the hotel industry only. Inclu…… (16:3498 [16:3661])-D 5: 12_Hussain et al. 2019.pdf**

Secondly, data have been collected from the hotel industry only. Including other service supply chains such as health care and banking may yield interesting insights.

**6:26 In the future, the proposed model can be extended to other ind…… (22:1992 [22:2335])-D 6: 18_Cherrafi et al. 2019.pdf**

In the future, the proposed model can be extended to other industrial sectors and manufacturing firms where the need to improve environmental performance is critical. The model can also be modified to extend its scope for reducing other environmental impacts and exploring the integration of more advanced techniques and tools.
